# CRISP: Cremated remains inference of sex probabilities – A software for Bayesian sex estimation in human cremated remains

**DOI:** 10.1371/journal.pone.0346813

**Published:** 2026-05-05

**Authors:** Lukas Waltenberger, Sophie Beitel, Mattia Bischeri, Katharina Rebay-Salisbury, Alessandra Sperduti, Claudio Cavazzuti

**Affiliations:** 1 Department for Prehistory and Historical Archaeology, University of Vienna, Vienna, Austria; 2 Austrian Archaeological Institute, Austrian Academy of Sciences, Vienna, Austria; 3 Center for Human Bioarchaeology and Paleogenetics, Faculty of Medicine, Vilnius University, Lithuania; 4 Christoph Beitel IT-Services, Vienna, Austria; 5 Centro di Archeologia per le Diversità e le MObilità preromane (CADMO), Università per Stranieri di Siena, Siena, Italy; 6 Human Evolution and Archaeological Sciences (HEAS), University of Vienna, Austria; 7 Servizio di Bioarcheologia, Museo delle Civiltà, Roma, Italy; 8 Dipartimento Asia, Africa e Mediterraneo, Università degli Studi di Napoli “L’Orientale”, Napoli, Italy; 9 Department of History and Culture, Alma Mater Studiorum-Università di Bologna, Bologna, Italy; 10 Istituto di Scienze per il Patrimonio, CNR-Consiglio Nazionale delle Ricerche, Roma, Italy; University of California Santa Cruz, UNITED STATES OF AMERICA

## Abstract

Sex assessment in cremation contexts represents a key step for reconstructing funerary rituals and demographic profiles. However, the high degree of fragmentation and thermal alteration of skeletal elements significantly hampers this operation. Most often, the morphological traits of pelvis and skull are not preserved while the application of metric criteria follows those developed from modern reference series. We used a prehistoric Italian sample (*n* = 155) with gender specific grave goods as a proxy for sex and developed binary logistic regression and Bayesian models based on 21 postcranial metric variables. We compared the results with the cut-off point method and validated them using an independent Austrian prehistoric sample (n = 45) sex estimation based on morphological sex traits. The Bayesian model achieved the highest accuracy (89%), outperforming cut-off points and regression models (68–88%, depending on the variable). In the Austrian sample, cut-off points produced the highest proportion of classified individuals but misclassified four times more cases than the other methods. Bayesian and regression models yielded higher rates of ambiguous classifications but maintained low error rates. Inconsistencies between the Bayesian sex prediction and morphological sex estimation can be explained by less reliable morphological traits in the morphological assessments. We also present CRISP, a new open-source software using the Bayesian approach for sex prediction. Multivariate statistical methods proved both more reliable and more adaptable for sex estimation in human cremated remains than univariate cut-off approaches. Validation demonstrated that metric methods substantially increase the number of individuals for which sex can be estimated and retain robustness when morphological indicators are ambiguous. The tool can further be used to identify deposits of cremated human remains composed of more than one individual. Minimal interpopulation differences between the Italian training and Austrian validation samples suggested CRISP is suitable for both Mediterranean and Central European contexts.

## Introduction

The identification of anthropological parameters, such as sex and age-at-death is a crucial component in bioanthropology and bioarchaeology. Sex estimation is integral to the reconstruction of osteobiographies and biological identities of individuals, as well as the understanding of funerary rituals, demographic structure, and the social organization of past communities. While more or less robust methods do exist for the analysis of unburnt skeletal remains [1], the analysis of cremated human remains is often difficult to perform and precarious due to extensive fragmentation, shrinkage, warping, and other heat-induced changes of bones [2–5]. These limitations often result in a greater number of undiagnosed individuals and higher error rates compared to unburnt skeletal remains [5].

Sex estimation in human cremated remains traditionally relies on either the assessment of sexually dimorphic morphological traits—primarily of the pelvis and cranium—or on metric approaches based on discriminant functions derived from single bone measurements [6]. Although morphological methods are generally preferred by anthropologists [7] as their validity has been extensively tested on unburnt skeletal samples, their application to cremated remains is severely constrained by fragmentation. Cremation frequently results in the partial or complete destruction of the most dimorphic skeletal elements, particularly the pelvis and skull [8–10]. Moreover, even when sex-diagnostic traits are observable, heat-induced deformation, delamination, and dimensional reduction substantially alter their morphology, causing cremated bones to appear more gracile than their unburned counterparts and increasing the risk of misclassification, particularly of male individuals.

As for the most metric methods, researchers have developed correction factors to handle these shrinkage effects, while applying cut-off points or discriminant functions developed from unburnt series. Metric approaches face similar limitations. Bone shrinkage induced by heat can reach up to 30% of the original dimensions, as demonstrated experimentally on ovine bones [11], and varies considerably according to skeletal element, bone composition, and burning conditions [12–14]. Significant shrinkage has been documented at temperatures exceeding 700°C, corresponding to complete calcination, whereas bones exposed to lower temperatures tend to retain dimensions closer to those of unburnt material [15]. This tends to increase the number of misclassified males. Earlier studies attempted to compensate for these effects by applying generalized correction factors (e.g., 10%) to measurements prior to using discriminant functions developed on unburnt samples [16]. However, such approaches oversimplify a process characterized by marked variability.

More recent research advocates chemosteometric approaches, which may provide greater accuracy than uniform correction factors, particularly in cases where shrinkage is highly irregular [15]. These developments underscore the need for metric sex estimation methods specifically calibrated on completely calcined human remains, in order to improve comparability and minimize bias introduced by heterogeneous burning conditions. Applying correction factors to adapt methods developed on unburnt skeletal material remains problematic, as sexual dimorphism already contributes substantial variability to prediction models, which is further amplified by element-specific and non-linear shrinkage during cremation. At present, the reliability of such corrected methods is difficult to assess and would require systematic testing on identified reference collections of cremated individuals.

Several studies have proposed sex estimation methods based on modern cremated remains [16–18]. While these approaches are valuable for forensic contexts, their applicability to archaeological cremation burials is limited as ancient populations are often biologically distinct (e.g., in terms of body mass) from the modern reference samples on which these methods are developed. Modern cremations are performed in highly controlled settings, using industrial furnaces with regulated oxygen supply and consistently high burning temperatures [19]. In contrast, prehistoric and ancient cremations were typically carried out on open-air wooden pyres, characterized by a heterogenous and fluctuating thermal conditions, variable pyre management, diverse funerary practices, and complex post-depositional processes [20–22].

For these reasons, sex estimation is often not possible for a substantial proportion of individuals recovered from archaeological cremation burials. Reported sex estimation rates vary widely across sites, depending on the study and the preservation of the remains, and range between 3% and 40% [7,23,24]. This highlights the need for further development of dedicated methods and analytical strategies specifically tailored for ancient cremations.

Recent studies have suggested potential methodological pathways to overcome, or at least reduce, these limitations. Among these, Cavazzuti, Bresadola [25] proposed a novel method developed on Bronze and Iron Age cremation contexts from Italy, employing gender-specific grave goods as proxies for sex. The study established cut-off points for 21 metric variables from various postcranial bones. The results demonstrated the persistence of high sexual dimorphism for the majority of the variables and moderate to good accuracy for 18 of them. This finding suggests that gender functions as a good proxy for sex, at least in this particular context. The method was subsequently validated through its application to archaeological cremated remains [18,26–28]. Particularly, the study of Bischeri, Alhaique [28] tested the correspondence between sex estimations, based on Cavazzuti et al.’ s metric method implemented by morphological observations, and typically gendered archaeological materials from the Etruscan sepultures of Tolle. In addition to grave goods (weapons for males; ornaments and spinning/weaving tools for females), gender was also indicated by the sexual attributes represented in the canopies (ossuaries shaped as a human face and body). Biological sex, estimated by osteologists, and archaeological gender, marked by grave goods and vessel features, matched in 95.6% of the cases.

The literature indicates that the majority of metric sex estimation methods in human cremated remains are predicated on statistical prediction techniques, including binary logistic regression, discriminant function analysis, and cut-off points (see [17,23,25]). These methods demonstrate accuracies ranging from 80 to 90%. Recently, the employment of advanced statistical methods, including machine learning algorithms, has been demonstrated to enhance accuracy to over 90% [18]. However, the application of these methods is challenging for researchers lacking familiarity with these intricate statistical techniques and programming. Consequently, they remain infrequently used in practice. A recent survey by Hlad, Löffelmann [7] revealed a lack of standardized practices in the osteological analysis of human cremated remains. The majority of practitioners rely on morphological assessments with minimal training in cremation-specific methods. Levels of confidence are notably low, particularly in the context of sex estimation, and the utilization of metric or computational approaches is limited. Given the lack of training opportunities in the analysis of human cremated remains and the significant experience and time required for professional analysis, there is an urgent need for simple and replicable methods of estimating sex of human cremated remains. Such methods should be applicable with adequate accuracy, even by researchers with limited experience. The objective of this study is to present and validate CRISP, a software for metric sex estimation based on Bayesian statistics. Such approaches have been utilized with success in the past, as evidenced by the DSP software for a metric sex estimation based on pelvic measurements [29]. The objective of this study is to present a method that is both highly accurate and user-friendly, thereby facilitating the analysis of human cremated remains also for researchers, who are not well-versed in these methods and computer coding.

## Material

The development of the Bayesian method was based on the compilation and analysis of previously published osteometric data from cremation burials at several Italian necropolises, dating from the Final Bronze Age to the Iron Age. These sites include the Narde di Frattesina necropolis in Veneto (Final Bronze Age, 12^th^-9^th^ century BCE; [30,31]); Chiavari, Liguria (Iron Age, 7^th^-6^th^ century BCE, [32]) Castenaso, Madonna del Buon Consiglio, Emilia Romagna (Iron Age-Villanovian: 7^th^ century BCE, [28]); Narce, Lazio (Iron Age-Faliscan, 8^th^-7^th^ century BCE, [33]); and Pontecagnano, Campania (Iron Age: 8^th^-7^th^ century BCE, [34]). These osteometric data were published in S1 in [25] and contain information on 124 individuals (50 males and 74 females). The osteological material and documentation are currently stored at the Bioarchaeology Service at the Museo delle Civiltà in Rome. Additionally, we included data from 31 individuals (16 males and 15 females) obtained from the Etruscan necropolis in Tolle, Tuscany (8^th^-5^nd^ century BCE, [28]). The inclusion criteria were single burials of adult individuals (complete skeletal development), bone color ranging from grey to calcined white, absence of osteoarthritic lesions or other pathological lesions that could affect the measurements, and gender-specific urn shapes and/or grave goods. Cavazzuti, Bresadola [25] based their gender estimations on grave goods – such as weapons and razors for men, and spindle whorls, simple-arch or ‘leech’ fibulas, and faience or glass beads for women. In Tolle, the gender of individuals was inferred from the design of canopic urns featuring anthropomorphic characteristics. These urns depict an idealized representation of the deceased, often including indicators of rank, jewelry, clothing, and, in many cases, gender-specific iconography [28].Additionally, we tested the applicability of the diagnostic method to Central European populations using a sample from the Late Bronze Age Urnfield Culture in Austria. The dataset included 45 individuals with osteometric data from the following sites in Lower Austria: St. Pölten Fuhrmannsgasse (14^th^ century BCE, [22]); Getzersdorf (13^th^-12^th^ century BCE, [35,36]); Inzersdorf (12^th^-10^th^ century BCE, [37–39]); and Franzhausen-Kokoron (10^th^-8^th^ century BCE, [40,41]). These sites were subjected to osteological analysis as part of the “Unlocking the Secrets of Cremated Human Remains” project. All necessary permits were obtained for the described study, which complied with all relevant regulations. The Federal Monuments Authority of Austria and Natural History Museum of Vienna granted access to the cremated remains from Austria and approved this study from an ethical perspective. The use and access of published data by Cavazzuti, Bresadola [25] is granted by the Bioarchaeology Service at the Museo delle Civiltà in Rome (Italian Ministry of Culture). Access to the Additional information regarding the ethical, cultural, and scientific considerations specific to inclusivity in global research is included in the Supporting Information (S4 Checklist).

## Methods

**Fig 1:**
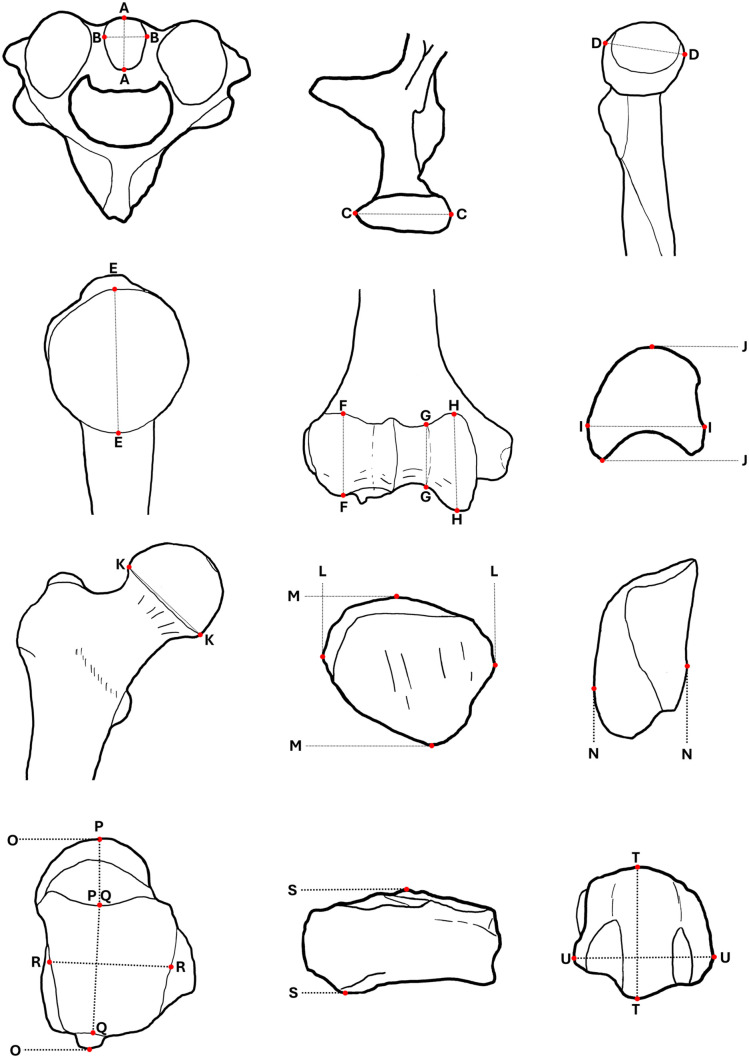
Measurement definitions for all variables. A-A: Axis: anterior-posterior diameter, B-B: Axis: transverse diameter, C-C: Mandible: condyle width, D-D: Radius: maximum head diameter, E-E: Humerus: vertical head diameter, F-F: Humerus: capitulum maximum diameter, G-G: Humerus: trochlea minimum diameter, H-H: Humerus: trochlea maximum diameter, I-I: Lunate: maximum width, J-J: Lunate: maximum length, K-K: Femur: vertical head diameter, L-L: Patella: maximum width, M-M: Patella: maximum height, N-N: Patella: maximum thickness, O-O: Talus: maximum length, P-P: Talus: head-neck length, Q-Q: Talus: trochlea length, R-R: Talus: trochlea width, S-S: Navicular: maximum length, T-T: MT1: dorsoplantar width of the head, U-U: MT1: mediolateral width of the head.

Fig 1 provides an overview of the measurement definitions of the variables utilized. The analysis was executed in R-statistics 4.4.1 by using the packages boot 1.3–30 [42], caret 6.0–94 [43], dplyr 1.1.4 [44], ggplot 2 3.5.1 [45], LogicReg 1.6.6 [46], rstatix 0.7.2 [47], tidyverse 2.0.0 [48], verification 1.42 [49], and visreg 2.7.0 [50]. Initially, t-tests were employed to identify potential group differences between the sexes, thereby identifying variables exhibiting significant sexual dimorphism. Furthermore, we calculated Chakraborty & Majumder D-values [51] to assess the nonoverlap of the distributions between both sexes per variable. Variables exhibiting significant sexual dimorphism were incorporated into a prediction model for sex.

To accomplish the objective, the emphasis was placed on three distinct approaches: Initially, binary logistic regression models were calculated as an extension of the cut-off points published in Cavazzuti, Bresadola [25]. In general, simple cutoff criteria facilitate the establishment of binary classification of sex based on a threshold value. However, these cutoffs do not encompass the complete distribution of measurements observed within each designated sex. Thus, individuals with values approximating the cut-off values are at elevated risk of misclassification. Conversely, binary logistic regression models incorporate these sex-specific distributions and estimate the probability that an individual belongs to a specific sex based on the observed measurement, offering a more nuanced and probabilistic approach to sex estimation.

A second approach to sex estimation involved the combination of multiple measured variables predicated on the premise that each variable reflected an aspect of sexual dimorphism. The integration of these variables has been demonstrated to improve classification accuracy. However, given the inherent fragmentation of archaeological cremation burials, multivariate models required a high degree of flexibility, as the complete documentation of all 21 target variables within a single burial was exceptionally rare. It is important to note that, generally, multiple binary logistic regression models and many other multivariate statistical models cannot manage missing data. In such instances, the imputation of missing values is imperative for the subsequent estimation of sex. Given that in our sample most of the individuals yielded one to three measurements out of the 21 method-defined variables, the final prediction may rely heavily on imputed values rather than actual observations. This has the potential to increase the risk of overfitting and reduce the model’s reliability.

Consequently, we developed a Bayesian model for sex prediction, offering both high accuracy and flexibility. To develop the method, we used a training set containing the recorded measurements from the Italian sample. The Bayesian model estimated the distributions of each variable based on this training dataset and calculated individual likelihoods for being male or female. As prior sex ratio we used equal priors for males and females (1:1) which reflects the sex ratio in adults. We also tested slightly skewed priors of 1.05:1 which corresponds to the natural sex ratio at birth. Comparison of the resulting posterior probabilities and classification outcomes showed only negligible differences in sex estimation accuracy between these prior settings, indicating that model predictions are primarily driven by the observed data rather than by prior assumptions. These measurements were then amalgamated into a posterior probability, thereby enabling the estimation of sex estimation even in circumstances where only a subset of the measurements was available. The Bayesian model employs distributions of the variables derived from a training set and assigns relative weights to the individual probabilities of each variable for sex estimation based on the probability distributions learned from the training set and subsequently integrating these probabilities to formulate a posterior prediction probability. A notable benefit of the Bayesian approach is its capacity to accommodate uncertainty and generate probabilistic classifications rather than binary outputs. This is of particular value in archaeological contexts, where data are often incomplete. In essence, the Bayesian model has the capacity to integrate measurements from one to 21 traits while concurrently providing a probabilistic sex classification. The predictive efficacy of the model is known to enhance as more measurements become available. Moreover, prior knowledge, such as expected sex ratios or population-specific distributions, can be integrated, enhancing the model’s relevance and interpretability and allowing for straightforward model updates in the future with increased data sets. The utilization of Bayesian models necessitates a foundation of statistical knowledge and proficiency in a programming language, such as R, Python, to execute the code. To address this need, we integrated the Bayesian model into an application. The model was updated in Python (Pycharm 2022.2.2, libraries: matplotlib 3.10.3, [52]; numpy 2.2.6, [53]; openpyxl 3.1.5, [54]; pandas 2.3.0, [55]; pathlib 1.0.1, [56]; PyQt6 6.9.1, [57]; scipy 1.16.0, [58]; and seaborn 0.13.2, [59]). In addition, an user interface was developed. The application can be downloaded and utilized offline. Beyond the capacity to predict individual cases, the application possesses the functionality to accommodate multiple case predictions derived from Excel spreadsheets.

The sex estimation models were evaluated using a leave-one-out cross-validation approach. The accuracy of the binary logistic regression models, the Bayesian models and the cut-off points (for which cut-off point accuracies were published in Cavazzuti, Bresadola [25]) were evaluated. In addition to reporting the raw classification accuracies, post-hoc filtering was also evaluated. In general, accuracy is calculated as the proportion of correctly classified individuals out of all cases using a default cut-off of 50% probability. However, in the discipline of osteology, it is customary to adopt a more cautious approach and to abstain from making sex estimation when the predicted probability approaches 50%, as this practice carries a high risk of misclassification and yields results that are nearly random. This approach has the potential to be misleading, if individuals with ambiguous sex estimates are included in the accuracy assessment [9,60]. There are no universally accepted thresholds for determining the probability required to confidently estimate sex in osteology. Consequently, an evaluation was conducted using five artificial post-hoc filtering thresholds (50%, 65%, 75%, 85%, 90%). Using post-hoc filtering as prediction thresholds minimizes prediction errors [61,62]. Individuals whose predicted probabilities did not exceed these thresholds were defined as “ambiguous” and excluded from further classification. For each threshold, classification accuracy was then recalculated based only on the remaining, non-ambiguous individuals. This approach enabled a more comprehensive evaluation of the models’ performance, with a particular focus on metrics such as balancing accuracy and confidence. Reliability curves were calculated for the Bayesian model and the ROC (Receiving Operating Characteristic) for the binary logistic regression models. Finally, we further validated the sex estimation models through the use of an independent test-set containing a sample from the Urnfield Culture in Austria. In contrast to the Italian sample, the availability of gender-specific grave goods was limited. The focus therefore shifted to a morphological sex estimation approach, which was based on the morphological expression of features at the cranium and pelvis as a proxy for sex [63]. The postcranial measurements obtained were then utilized for sex prediction purpose, employing sex estimation models that were developed based on the Italian sample.

## Results

The distribution of the Italian and Austrian samples is presented in Table 1. The dataset, including measurements recorded for each individual is provided in S1 Data (.csv). Interobserver errors and technical error of measurements (TEM) of the variables have been published in Cavazzuti, Bresadola [25]. All used variables have low relative technical errors of measurements (<0.05). With the exception of the transverse diameter of the axis and the head-neck length of the talus, all variables showed significant differences between males and females and were included into the sex prediction models (see Table 2). Notably, the measurements of the mediolateral head width of the first metatarsal were found to be not normally distributed. In accordance with the assumptions underlying the Bayesian model, which requiresnormally distributed data, the measurements were log-transformed. The measurement of the humerus trochlea maximum diameter of case 149 (Tolle grave 116, male individual) was excluded from further analysis. This measurement was an outlier and the lowest of all cases. Given that other measurements of this individual were in the top range of the male sample, this value was suspected to be a measurement error.

**Table 1:** Data set presenting the distribution of the metric variables of the Italian and Austrian samples. The Italian sample was used to develop the sex estimation models based on gender information. The Austrian sample was based on morphological sex estimation and it was used for validation. The data sets present the results for males and females separately.

variable	sex/ gender^1^	Italian sample					Austrian sample				
*n*	mean	median	sd	range	*n*	mean	median	sd	range
Mandible: condyle width	M	24	16.95	16.68	1.44	14.92-20.54	4	16.69	16.48	0.64	16.20-17.61
	F	19	14.77	14.59	1.58	10.70-17.67	3	14.32	14.63	1.66	12.53-15.80
Axis: ant.-post. diameter	M	34	9.97	10.01	0.94	8.27-12.62	5	9.85	9.89	0.83	8.96-10.86
	F	26	8.90	9.02	0.80	7.19-10.5	6	9.05	9.23	0.79	7.62-9.82
Axis: transv. diameter	M	33	8.97	8.96	0.77	7.68-11.39	2	9.32	9.32	1.61	8.18-10.45
	F	26	8.83	8.87	0.66	7.53-10.5	3	8.13	7.71	1.24	7.15-9.52
Humerus: vert. head diameter	M	14	40.63	41.17	1.67	36.5-45.69	2	40.92	42.92	2.85	38.91-42.93
	F	19	35.50	35.74	2.20	31.73-38.94	1	40.75	40.75	–	40.75-40.75
Humerus: trochlea max. diameter	M	15	20.77	20.96	2.33	18.00-25.53	1	18.37	18.37	–	18.37-18.37
	F	20	18.67	18.45	1.36	16394-21.52	0	–	–	–	–
Humerus: trochlea min. diameter	M	28	13.75	13.61	1.72	10.92-17.70	4	13.32	13.21	0.59	12.74-14.12
	F	34	12.14	11.98	1.29	9.94-15.05	1	13.70	13.70	–	13.70-13.70
Humerus: capitulum max. diameter	M	12	17.07	17.31	1.33	14.69-18.68	4	17.44	17.25	1.06	16.38-18.88
	F	16	15.60	15.18	1.93	12.41-20.76	0	–	–	–	–
Radius: max. head diameter	M	27	19.77	19.86	1.29	15.81-22.02	3	19.15	19.42	0.65	18.41-19.63
	F	39	17.07	17.10	1.28	14.65-20.25	1	15.29	15.29	–	15.29-15.29-
Lunate: max. width	M	13	14.73	15.17	1.32	12.26-16.68	2	15.79	15.79	2.57	13.97-17.61
	F	12	13.23	12.74	1.28	11.54-15.41	0	–	–	–	–
Lunate: max. length	M	9	14.23	14.16	1.12	12.62-15.89	3	14.71	15.66	1.65	12.81-15.67
	F	17	12.01	12.54	2.10	6.50-14.80	1	11.34	11.34	–	11.34-11.34
Femur: vert. Head diameter	M	11	42.14	42.47	3.15	36.07-46.67	0	–	–	–	–
	F	17	36.24	36.70	3.44	29.08-41.85	1	38.40	38.40	–	38.40-38.40
Patella: max. height	M	10	38.98	39.20	2.17	35.56-42.47	0	–	–	–	–
	F	15	34.61	34.67	1.46	31.47-36.70	0	–	–	–	–
Patella: max. width	M	8	37.73	36.73	3.38	33.59-43.28	0	–	–	–	–
	F	15	33.76	34.43	2.35	28.70-37.00	1	34.00	34.00	–	34.00-34.00
Patella: max. thickness	M	25	16.61	16.67	2.24	12.34-20.56	4	17.11	16.73	–	16.00-18.96
	F	42	14.66	14.53	1.73	11.53-18.90	2	14.19	14.19	2.57	12.37-16.00
Talus: max. length	M	6	48.84	48.34	2.41	46.14-52.24	0	–	–	–	–
	F	11	44.93	44.00	2.50	41.75-50.00	0	–	–	–	–
Talus: head-neck length	M	9	17.70	19.16	4.05	9.27-21.82	0	–	–	–	–
	F	13	17.27	18.18	3.65	9.33-20.91	0	–	–	–	–
Talus: trochlea length	M	13	31.52	31.24	2.37	28.38-35.91	0	–	–	–	–
	F	16	26.87	26.80	2.18	21.65-30.85	0	–	–	–	–
Talus: trochlea width	M	24	29.70	29.18	2.91	24.51-35.11	0	–	–	–	–
	F	34	25.98	26.32	2.29	20.67-29.68	0	–	–	–	–
Navicular: max. length	M	15	14.00	14.07	2.46	9.28-17.79	0	–	–	–	–
	F	23	11.94	11.65	1.61	8.08-15.92	0	–	–	–	–
MT1: dorsoplantar width of the head	M	28	17.23	17.12	1.42	14.63-20.13	4	18.11	17.57	1.38	17.20-20.11
	F	35	15.28	15.33	1.29	11.80-17.38	1	17.00	17.00	–	17.00-17.00
MT1: med.-lat. width of the head	M	22	18.88	18.48	1.90	15.84-22.56	2	17.07	17.07	0.04	17.04-17.10
	F	27	16.33	16.16	1.67	13.32-20.48	3	17.35	17.10	0.57	16.94-18.00

**¹** The Italian sample was classified into male and female categories based on gender inferred from gender-specific grave goods, whereas the Austrian sample was classified based on biological sex determined from skeletal remains.

**Table 2:** Differences between the male and female subgroups for each variable based on the results of student’s t-tests and Chakraborty and Majumder’s D-values.

variable	*n*_male_	*n*_female_	mean_male_	mean_female_	student’s t	p-value	Chakraborty and Majumder’s D-values
Mandible: condyle width	24	19	16.95	14.77	4.45	**<0.001**	0.53
Axis: ant.-post. Diameter	34	26	9.97	8.9	4.63	**<0.001**	0.46
Axis: transverse diameter (log)	33	26	2.19	2.18	0.75	0.455	0.10
Humerus: vert. head diameter	14	19	40.63	35.50	6.00	**<0.001**	0.71
Humerus: trochlea max. diameter	15	20	20.77	18.67	4.10	**<0.001**	0.52
Humerus: trochlea min. diameter	28	34	13.75	12.14	4.23	**<0.001**	0.42
Humerus: capitulum max. diameter	12	16	17.07	15.60	2.28	**0.031**	0.38
Radius: max. head diameter	27	39	19.77	17.07	8.42	**<0.001**	0.71
Lunate: max. width	13	12	14.73	13.23	2.87	**0.009**	0.44
Lunate: max. length	9	17	14.23	12.01	2.93	**0.007**	0.55
Femur: vert. Head diameter	11	17	42.14	36.26	4.57	**<0.001**	0.52
Patella: max. height	10	15	38.98	34.61	6.03	**<0.001**	0.78
Patella: max. width	8	15	37.73	33.76	3.32	**0.003**	0.53
Patella: max. thickness	25	42	16.61	14.66	4.00	**<0.001**	0.39
Talus: max. length	6	11	48.84	44.93	3.12	**0.007**	0.57
Talus: head-neck length	9	13	17.70	17.27	0.26	0.796	0.05
Talus: trochlea length	13	16	31.52	26.87	5.49	**<0.001**	0.69
Talus: trochlea width	24	34	29.70	25.98	5.46	**<0.001**	0.52
Navicular: max. length	15	23	14.00	11.94	3.12	**0.004**	0.41
MT1: dorsoplantar width of the head	28	35	17.23	15.28	5.69	**<0.001**	0.53
MT1: med.-lat. width of the head (log)	22	27	2.93	2.79	5.08	**<0.001**	0.54

### Validation

**Fig 2:**
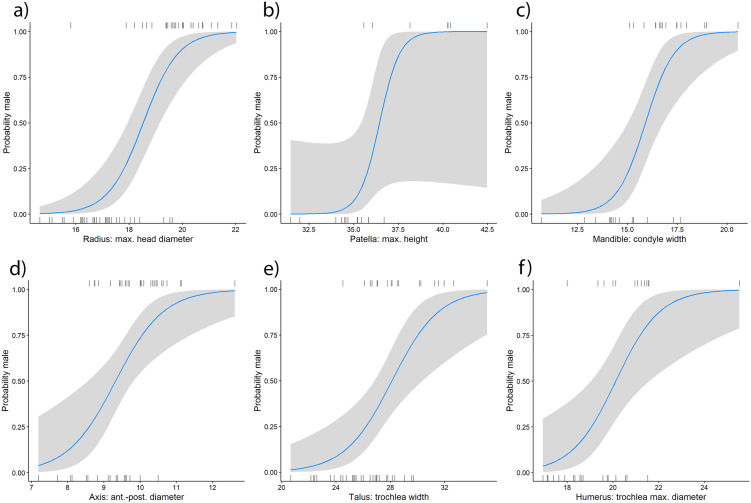
Probability curves for sex prediction of the logistic regression models of the variables with the highest accuracies (a-c) and lowest accuracies (d-f). The presence of vertical lines at the bottom and top of the plots indicates the measurement values of individual cases, with gray areas denoting the 95% confidence intervals. **a)** Radius: maximum head diameter, **b)** Patella: maximum height, **c)** Mandible: condyle width, **d)** Axis: anterior-posterior diameter, **e)** Talus: trochlea width, **f)** Humerus: trochlea maximum diameter.

The accuracies of the cut-off point method published by Cavazzuti, Bresadola [25] had been derived by dividing their dataset randomly in a training and a validation subset. The accuracy for the binary logistic regression models and the Bayesian model was estimated using a leave-one-out cross-validation (LOOCV). The accuracies obtained for each variable prior to post-hoc filtering (hereafter referred to as raw accuracies), of the cut-off points and the binary regression model, were similar. The cut-off point method yielded the highest raw accuracies for the variable radius head maximum diameter (88%). The maximum width of the patella (86%), and the width of the mandibular condyle (84%) also demonstrated high levels of accuracy. The anterior-posterior diameter of the axis, maximum height of the patella, and humerus trochlea minimum diameter exhibited the lowest raw accuracies (68%, 69%, and 72%, respectively). In the binary logistic regression models, the radius head maximum diameter, maximum height of the patella, and width of the mandibular condyle demonstrated good performance with respective accuracies of 88%, 86%, and 82%, as illustrated in Fig 2a-c. Conversely, the anterior-posterior diameter of the axis, trochlea width of the talus, and humerus trochlea maximum diameter exhibited the lowest raw accuracies with percentages of 68%, 70%, and 71%, respectively, as shown in Fig 2d-f. Detailed results of the accuracies per variable and method can be found in S1 File.

The cut-off-point method and the binary logistic regression method yielded comparable per-variable raw accuracies averaged across all univariable models (cut-off point: 75%, regression models: 77%). Multivariable accuracy estimates were not calculated for the binary logistic regression approach due to missing data constraints, whereas multivariable integration was performed using the Bayesian model. As expected, the Bayesian model, which employed combinations of variables for sex prediction, demonstrated superior performance compared to the sex prediction based on single variables (raw accuracy 86%). The sensitivity and specificity of the binary logistic regression models and the Bayesian model indicate a greater number of correctly classified females (higher specificity than sensitivity). Subsequent to the implementation of post-hoc filtering (75% threshold), a marginal enhancement in the accuracy of both models was observed (Table 3).

**Table 3:** Performance of the classification measures for the binary logistic regression and Bayesian models. To refine model evaluation, subjects with a sex prediction probability below 75% were designated as ambiguous and excluded from the analysis (post-hoc filtering), thereby facilitating a more accurate depiction of model performance.

	binary logistic regression models		Bayesian model	
performance	raw	post-hoc filtering	raw	post-hoc filtering
accuracy	76.9	83.9	85.6	88.9
sensitivity	69.0	75.9	81.8	85.7
specificity	81.5	87.1	88.5	91.4
precision	73.79	75.0	84.4	88.9
F1-score	71.12	76.5	83.1	97.3

**Fig 3:**
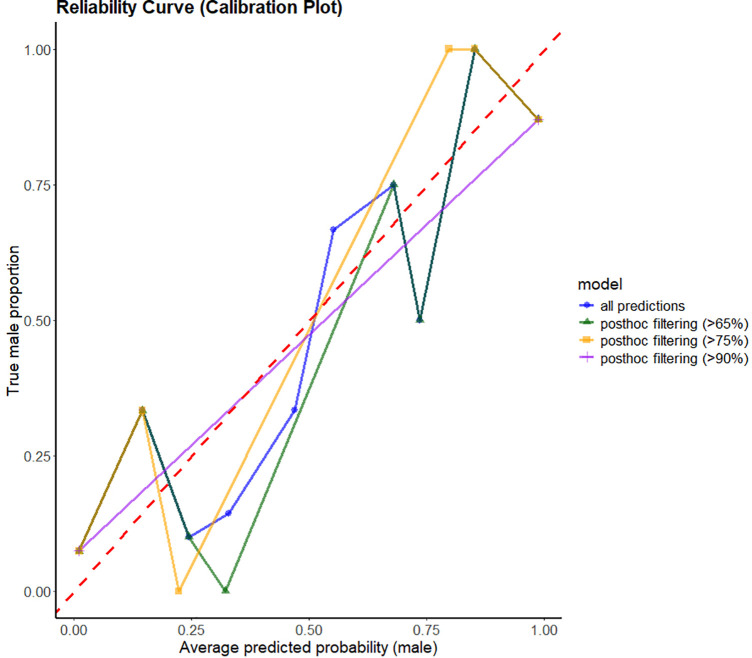
Calibration plot encompassing the reliability curves of the Bayesian model and post-hoc filtering with varying thresholds. The validation study exclusively included individuals with a predicted sex probability that exceeded the threshold. The red dotted line represents a technically perfect calibrated model. In practice, a model with a reliability curve below the red-dotted line in the area below the average probability of 0.5 and above the red-dotted line in the area above the average probability of 0.5 is ideal for distinguishing between females and males.

To this end, a series of post-hoc filtering thresholds were tested in order to exclude individuals with an ambiguous sex estimate from the model accuracy (thresholds: 65%, 75%, 85%, 90%). Overall higher thresholds did not result in a substantial increase in accuracy; however, they led to a significant number of cases being classified as ambiguous. For instance, in binary logistic regression models with a threshold of 75%, the exclusion of cases ranged from 9.5% to () and 57.5% depending on the variable (lowest rate of ambiguous estimated individuals: patella: maximum height; highest rate of ambiguous estimated individuals: dens axis: anterior-posterior diameter). This post-hoc filtering threshold of 75% resulted in an average increase in accuracy of 7% in the logistic regression models. In the Bayesian model, 19.6% of all cases were classified as ambiguous, with only a slight increase in accuracy of 3%. It has been demonstrated that alternative post-hoc filtering thresholds (65%, 85%, 90%) exhibit comparable classification performance, with accuracies fluctuating between 87 and 90%. Furthermore, calibration curves were calculated for different thresholds to assess the quality of the sex prediction in greater detail (Fig 3 and Table 4). Generally, the calibration lines indicated good calibration of the Bayesian model for sex prediction. The calibration line without post-hoc filtering indicated good calibration in the lower and middle regions of the plot. However, it exhibited an over proportional number of female estimates compared to the expected numbers. Post-hoc thresholds yielded comparable outcomes and exhibited better calibration, with enhanced prediction accuracy for male subjects.

**Table 4:** Different thresholds for post-hoc filtering utilized for the Bayesian models along with the number of cases that fall into the ambiguous category. These cases were subsequently excluded from a successful sex estimation.

threshold [%]	–	65	75	85	90
*n*_*male*_	66	59	56	48	42
*n*_*female*_	87	81	67	60	56
percentage decrease in sample size between groups	0	8.5	12.1	12.2	9.3
percentage decrease relative to total sample size	0	8.5	19.6	29.4	35.9
accuracy	85.6	87.4	88.9	90.0	90.0

In conclusion, the accuracies after post-hoc filtering were found to be similar, with a negligible increase in accuracy in higher thresholds but a large increase of ambiguous classified cases from 9 to 36%. The 65% post-hoc filtering threshold had been determined to offer an optimal compromise, ensuring both high accuracy and a substantial number of cases with successful sex estimates. In other words, we suggest to interpret sex prediction probabilities obtained in CRISP lower than 65% as ambiguous and only assign a sex prediction to individuals with a prediction probability of 65% or more.

### Application CRISP: Cremated Remains Inference of Sex Probabilities

**Fig 4:**
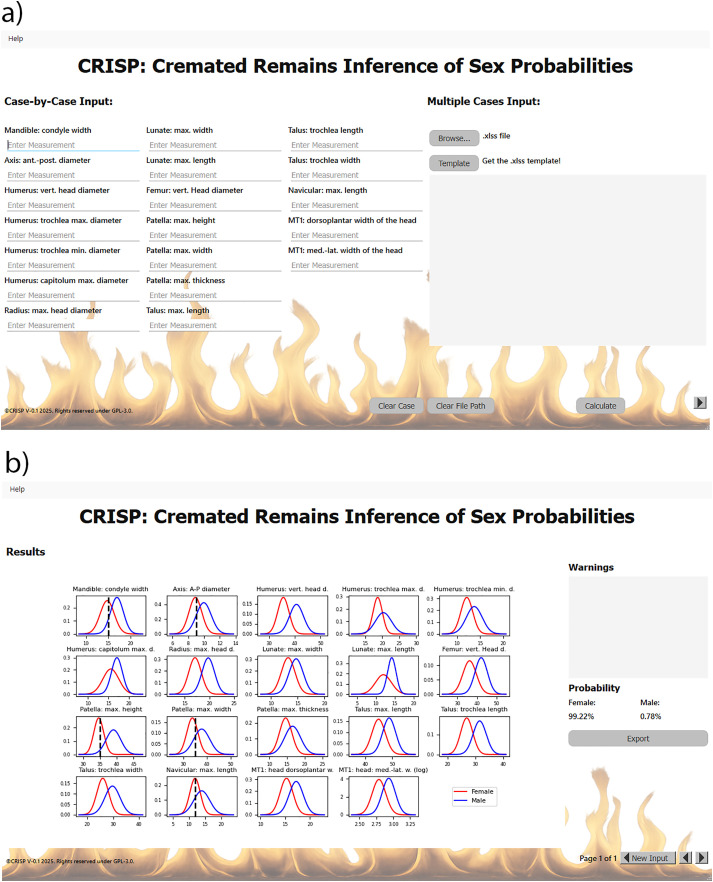
CRISP software interface. **a)** Main page of the software for data recording.The available measurements for individual cases can be documented in the boxes located on the left side of this page. The estimation of multiple cases can be achieved through the upload of all relevant recordings into an Excel spreadsheet. Please use the provided template (button *template*). The file can be important into CRISP by selecting the *Browse* button. The calculation of the sex prediction is initiated by the act of pressing the designated button. **b)** Results page. The plots represent the data distribution (separated in females and males) used for the prediction model. The vertical dotted lines denote the individual measurements incorporated into the prediction. Right, you can find a box showing warnings, should they occur (see section “Warnings” for more details). Additionally, the results of the sex prediction are presented as probabilities, indicating whether the subject is male or female. The export of a report on the results for individual prediction is initiated by pressing the *Export* button. A detailed explanation can be found in the manual integrated into CRISP.

The code for the Bayesian model and the application software CRISP can be obtained from GitHub (https://github.com/quadraBits/CRISP, published under software license GPL-3.0). The application’s functionality extends to an offline mode, accompanied by a user-friendly interface (Fig 4a). At present, two options are available: a single case input, and a multiple cases input. CRISP allows for direct inscription of measurements in millimeters for elements originating from a single case. CRISP is a flexible tool that allows the inclusion of up to 21 measurements per individual for sex prediction. When multiple cases are to be analyzed, an additional option allows users to record all measurements in an Excel spreadsheet and to automatically predict sex of all cases simultaneously. The spreadsheet template can be accessed within the application. For the single prediction, a “result page” is provided, which contains density plots for all variables (Fig 4b). A black dotted line is visible to highlight the location of the predicted single measurements within the distribution curves for both males and females. This allows a first assessment to identify suspected measurement errors and outliers. Furthermore, the probability of the subject being a male or female will be displayed. CRISP does not provide any interpretations of the results (e.g., the individual is “possible male/female”, “probably male/female”, or “male/female”) This discrimination into sex-specific groups are artificial, and definitions vary slightly between researchers, research groups, and countries.

Recent research has underscored the challenges associated with the identification of multiple individuals interred within a single urn, despite the utilization of morphometric analysis (see [64]). Consequently, there is always some uncertainty the measurements obtained from different bone fragments may belong to the same or different individual. The combination of commingled elements of a male and female into a unified prediction may have an impact on the sex prediction. To avoid this, CRISP incorporates three distinct data control mechanisms. First, a warning will be issued if a measurement falls outside of three standard deviations of the variable distributions utilized for the Bayesian model. This will serve to underscore potential typographical errors and measurement inaccuracies. In instances where two to four measurements are employed for prediction, and the posterior prediction probabilities for at least two features deviate from each other by more than 33%, the warning “Inconsistency for few features!” is generated. This warning is accompanied by the discrepancy between the posterior prediction probabilities for the two features that deviate from each other. In the event that five or more measurements are utilized for sex prediction, and one or more observations yield contradictory results, the warning “Inconsistency for several features!” will be displayed. In this instance, the single posterior probabilities will be compared to the median posterior probability, and predictions with a deviation of more than 0.25 will be highlighted. In other words, when such warnings are triggered, they highlight substantial variation among the measurements, raising the possibility that the analyzed elements originate from more than one individual. We advise osteological practitioner to critically analyze the cremation burial a second time to conclude whether to keep all measurements for prediction or to reject single measurements due to a suspected second individual. It should be noted that bone morphology exhibits variation both between and within populations. Consequently, the aforementioned warning shall be regarded as mere indications, rather than definitive evidence, for the presence of multiple individuals. It is important to note that an alternative interpretation in certain cases is possible, if the sex prediction derived is affected by a deviating posterior prediction probabilities (e.g., a single posterior probability of 90% indicating male) and a second less discriminant measurement falling into the ambiguous range (e.g., a single posterior probability of 59% indicating male). In such a case the warning can be disregarded. In reality, those deviations usually have a negligible impact on the results of the sex prediction as the variables are weighted in the Bayesian model.

The results of single predicted cases can be exported as a PDF file containing all of the information that is presented on the results page. If the Excel spreadsheet containing data on multiple cases is used, the sex of each case will be predicted based on the information provided. The probabilities and warnings will be automatically documented in the provided Excel file for subsequent evaluation. Updated versions of CRISP will be made available on GitHub (https://github.com/quadraBits/CRISP). Detailed instructions to use CRISP can be found at the GitHub folder in the MANUAL.pdf. The manual is also directly accessible via the menu in CRISP software.

### Test with an independent sample

In order to assess the adequacy of the sex prediction model in other populations, an independent sample from the Late Bronze Age of eastern Austria was utilized as a case study. The human cremated remains from this sample were predominantly highly fragmented, with a median fragment size between 5–10 mm and a bone weight of less than 200 g in adults. A comprehensive analysis of substantial case series was conducted in the Unlocking the Secrets of Cremated Human Remains project, encompassing over 700 cases, with a subset of 457 adult individuals. A meticulous examination of these cases revealed that a mere 43 cases yielded anatomical features conducive to morphological sex estimation, but also at least one anatomical structure allowing measurement and thus metric sex estimation. It is noteworthy that seven cases were estimated ambiguous by morphological sex estimation, necessitating further scrutiny to ascertain their precise classification. A comparison was made between the consistency of the results of the sex estimation based on morphological features and the three metric methods (complete results in S1 Table).

The findings (Table 5) indicated that the cut-off point method developed by Cavazzuti, Bresadola [25] exhibited the highest proportion of correctly classified individuals (69.4%), yet they also concomitantly demonstrated the greatest number of misclassified individuals (22.2%). The use of cut-off points always produced a classification, resulting in binary outcomes without associated probabilities. The classification of individuals of being “ambiguous” (three cases) was applied exclusively in instances where the evaluation of multiple features resulted in contrary results. The single binary logistic regression and Bayesian model exhibited 52.8% and 55.6% correctly classified individuals, along with notably higher rates for individuals categorized as “ambiguous”. In contrast, the rates for individuals showing inconsistencies between morphological and metric sex estimation were considerably lower than those observed using the cut-off point method. In summary, the Bayesian model and binary logistic regression models appeared to be more conservative with ambiguous cases, whereas the cut-off point method yielded higher classification rates but came with a four-fold increased risk of misclassifying individuals.

**Table 5:** Comparison of the sex estimation based on the three metric methods and the morphological sex estimation.

	cut-off point		single log reg models		Bayesian model	
n	%	n	%	n	%
consistency between morphological and metric sex estimation	25	69.44	19	52.78	20	55.56
morphological sex estimation successful; metric sex estimation ambiguous	3	8.33	13	36.11	14	38.89
inconsistency between morphological and metric sex estimation	8	22.22	4	11.11	2	5.56
total number of cases	36	100	36	100	36	100

Table 6 provides information on cases that deviate from the established sex estimations based on morphological and metric variables. The findings indicated that most individual misclassifications by the cut-off point method involved measurements in proximity to the cut-off point or exhibiting variables of low accuracy. These individuals were also classified as “ambiguous” in both the logistic regression and Bayesian models. In addition, Inzersdorf grave 181 exhibited contradictory outcomes in the binary logistic regression. The radius head indicated a male individual (*p* = 98.9), while the humerus trochlea minimum diameter and the lunate width suggested a female individual (both: *p* > 99.99) suggesting the presence of a second individual or an admixture phenomenon. Furthermore, in cases where the Bayesian model or binary logistic regression models yielded inconsistent results for morphological sex estimation, the latter was typically performed on less reliable cranial features such as the supraorbital margin and ridge or nuchal crest. In such instances, we hypothesize that the reliability of morphological sex estimation is diminished, potentially leading to misclassification.

**Table 6:** Cases with inconsistent sex estimation results between the morphological and metric sex estimation methods (deviating results highlighted in gray). The sex was estimated as male/female (M/F, *p* ≥ 90), probably male/female (M?/F?, 80 ≤ *p* < 90), possible male/female (M??/F??, 65 ≤ *p* < 80), or ambiguous (A, 50 ≤ *p* < 65).

site	grave	sex estimation								morphological features	metric features
**morphology**	**cut-off points**	**binary logistic regression**			**Bayesian model**		
			**probability [%]**			**probability [%]**	
			male	female		male	female
Franzhausen-Kokoron	326	M?	F	A	41.0	59.0	A	35.92	64.1	greater sciatic notch	axis: ant.-post. diameter
Franzhausen-Kokoron	499	M?	F	F?	14.1	85.9	F??	21.42	78.6	supraorbital margin/ridge, glabella	humerus: trochlea min. & max. diameter
Franzhausen-Kokoron	560	M??	F	A	36.7	63.3	F??	32.52	67.5	supraorbital margin/ridge	axis: ant.-post. diameter
Franzhausen-Kokoron	566	F??	M	A	58.0	42.0	A	61.02	39.0	nuchal crest	humerus: trochlea min. diameter
Franzhausen-Kokoron	925	F	M	A	44.7	55.3	A	42.48	57.5	supraorbital margin/ridge, greater sciatic notch	MT1: med.-lat. width of the head
Franzhausen-Kokoron	950	F??	M	M??	67.8	32.2	A	61.84	38.2	supraorbital margin/ridge	axis: ant.-post. diameter
Getzersdorf	61	M?	F	A	40.8	59.2	A	50.15	49.9	nuchal crest, mastoid process	patella: max. thickness
Inzersdorf	181	M??	F?	F	0	100.00	M??	78.10	21.9	nuchal crest	humerus: trochlea min. diameter lunate: max. width, radius: max. head diameter
Inzersdorf	217	M??	M	F	0	100.00	A	62.65	37.4	supraorbital margin/ridge	patella: max. thickness

## Discussion

Estimating the sex of individuals from cremated remains is a critical component of demographic studies of cemeteries, also contributing to the reconstruction of funerary behaviors and osteobiographies. Given the challenges posed by cremation, it is essential to employ all available methods, integrating commonly used morphological observations with metric approaches based on the measurement of skeletal elements. However, due to the effect of shrinkage and a high degree of fragmentation, highly diagnostic morphological features are rarely available, especially those from the fragile pelvic bones, and sex estimates are therefore often not feasible and, even when obtainable, not always reliable. Moreover, this analysis strongly relies on the observer’s experience and may implicitly entail inter-observer or inter-laboratory error. In contrast, metric sex assessments can be used on very fragmented individuals.

In archaeological contexts, the use of gender-associated grave goods as a proxy for biological sex has been adopted as a pragmatic and methodologically justifiable approach. Grave goods provide insights into an individual’s social role and presumed gender identity, which may, though not invariably, correspond to biological sex. However, there is a risk of conflating biological sex with socially constructed gender roles [65]. Furthermore, the correlation between specific grave goods and gender may vary across cultures, time periods, and even within the same society, thereby complicating the formulation of broad generalizations. This is particularly salient in cultures where gender is conceptualized as fluid, or where determinants such as social status and age appear to have exerted greater influence on grave goods assemblages than biological sex. Moreover, the specific funerary practices and the social organization underlying gender-specific grave goods are often not fully understood, which can result in biased interpretations and datasets. The selection of funerary rites, including grave goods, is typically influenced by a multifaceted array of factors, including gender, age, social status and, in some cases, the circumstances surrounding the death. This selection process is further influenced by the personal interests of the deceased individual [66,67]. Nonetheless, the validity of Cavazzuti et al.’s approach is further substantiated by numerous archaeological case studies from prehistoric Europe, which demonstrate the prevalence of gender-specific funerary practices across various regions [68,69].

These general associations between biological sex and gender expression in inhumed individuals have also been confirmed using methods beyond osteological analysis, such as DNA and amelogenin peptide analysis. The latter has recently emerged as a cost-effective, rapid, and minimally invasive approach, showing high of accuracy comparable to that of DNA analysis [65,70–72]. Together, these methods allow for a more nuanced evaluation of gender identity and social role. With a few notable exceptions (citations), numerous studies have documented a strong correspondence between chromosomal sex and presumed gender identity inferred from grave goods, particularly within well-structured burial traditions [65,68,73,74].In Austrian and Iberian prehistoric archaeological contexts, the biological sex of deceased children has been shown to consistently corresponded to a gender-specific burial placement [68,74]. Overall, these studies suggest that the use of gender as a proxy for sex in the development of sex estimation methods for human cremated remains is a valid strategy, provided that it is applied to societies characterized by high gender intensity and that sexing criteria are developed using robust statistical tools.

As outlined above, heat-induced bone shrinkage depends on several interacting factors, including the original morphology and mass of the skeletal elements as well as the temperature reached during cremation. These variables can introduce substantial variability in the degree of volumetric reduction observed both across cremations and within skeletal elements of the same individual [11,15]. To reduce the effect of random variation related to differential shrinkage, the present study focused exclusively on fully calcined fragments. As demonstrated in previous studies, sexual dimorphism in postcranial measurements is retained even after heat-induced bone shrinkage. The use of dry bone material for sex estimation has been shown to yield similar discriminatory power that is comparable to those obtained from cremated remains [18,25,75]. It is evident that postcranial skeletal dimensions demonstrate interpopulation variability, a phenomenon attributable to a combination of genetic, environmental, and lifestyle factors. It is important to note that methods developed on modern identified collections may not fully capture the morphological characteristics of prehistoric groups. This is due to secular changes, differential activity patterns, population-specific sexual dimorphism and different cremation techniques and post-depositional processes, which can influence bone size and shape [76–78]. Consequently, the application of metric sex estimation models, which have been trained on contemporary datasets, to archaeological samples necessitates caution, as systematic differences may affect prediction accuracy. Therefore, methods, such as CRISP needs to be developed based on archaeological material directly. The validation of the CRISP model with the Austrian sample suggests its applicability to prehistoric populations in Central and Southern Europe; however, further validation studies utilizing data from other populations are required to provide a more comprehensive evidence base.

The accuracy of metric sex estimation in human cremated remains has traditionally relied on cut-off points applied to single osteometric variables. While such univariate approaches offer simplicity and are frequently used in dry bone material (e.g., [79–81], they often suffer from reduced discriminatory power due to sex overlap. Research has demonstrated that classification based on solitary threshold per variable frequently yielded lower accuracies – typically ranging from 65 to 80% - and is particularly susceptible to misclassification in proximity to the cut-off point [82]. The employment of sophisticated machine learning algorithms, in conjunction with post-hoc filtering mechanisms, has led to a marked enhancement in the precision of the models in comparison to the utilization of conventional cut-off points. This enhancement can be attributed to the capacity of probabilities to facilitate a more nuanced interpretation of the outcomes. Multivariate statistical models and machine learning algorithms demonstrated superior performance by integrating multiple sexually dimorphic traits and weighting their contributions more appropriately [29,83,84]. Even if these variables exhibited minimal sexual dimorphism, resulting in suboptimal accuracy (e.g., the dens axis), their integration could potentially enhance accuracy significantly. The application of a Bayesian model resulted in a notably high accuracy of 87–90% after post-hoc filtering. The classification of individuals with posterior sex probabilities below a threshold, for example, 65%, as ambiguous was undertaken to reduce the risk of misclassification and ensure greater diagnostic reliability. Such probabilistic frameworks are particularly advantageous for fragmented cremated remains, where variable availability is inconsistent. These findings underscore the efficacy of flexible multivariate approaches over rigid univariate classification schemes in osteological sex estimation, particularly within the context of taphonomic constraints associated with cremations. A significant challenge is that multivariate methods and machine learning are intricate techniques that necessitate computer programming proficiency to process and interpret the code that implements sex estimation, which is frequently disseminated alongside the findings. This may discourage some researchers lacking familiarity with programming, and diminishes the acceptance and implementation of these methodologies in research projects and osteological analyses of cremation burials in archaeology. Consequently, we decided to develop a software application with a user-friendly accessible frontend.

The validation of metric sex estimation methods frequently relies on morphological sex assessments, which remain the standard approach in bioarchaeology and forensic anthropology [85]. In this study, we evaluated the performance of both cut-off-based and probabilistic metric models against morphological classifications in a sample of human cremated remains from Bronze Age Austrian necropolises. Our findings confirmed that the osteometric approach is a viable method for sex estimation and it can also be applied to highly fragmented individuals, where morphological traits are poorly preserved or difficult to assess. Cut-off point methods frequently yielded results that deviated from morphological assessments, particularly in cases where values clustered near the threshold. Conversely, multivariate approaches, including binary logistic regression and the Bayesian model, demonstrated a higher degree of concordance with morphological classifications. Notably, discrepancies between the Bayesian model and morphological assessments were predominantly observed in cases where posterior probabilities were low or when the morphological features employed for sex estimation were themselves less reliable. Features such as the supraorbital margin or nuchal crest, despite their frequent utilization, were recognized for their diminished interobserver agreement and augmented sexual overlap [9]. These discrepancies underscored a pivotal interplay between trait reliability and model confidence. When probabilistic models deviate from morphological classifications, they frequently do so with appropriately reduced certainty. This can serve as an internal indicator for ambiguous or borderline cases. Consequently, instead of perceiving these mismatches as errors, such discrepancies might be indicative of inherent biological ambiguity or limitations in the available osteological data, particularly in cases involving cremated and fragmentary remains [25]. For example, it may lead to the detection of multiple individuals deposited in a single cremation deposit.

A total of 457 adult individuals were identified in the Austrian sample. However, 260 (56.9%) had neither morphological nor metric traits available for sex estimation. A total of 130 individuals (28.4%) were sexed using morphological analysis. In 59 cases, the morphological criteria were not applicable. However, at least one measurement for the Bayesian model was obtained, resulting in an increase of 12.9% in successfully estimated sex. Consequently, the implementation of both approaches would facilitate the identification of the sex of approximately 40% of the individuals. This outcome is particularly noteworthy, considering the fragmentation of the skeletal material, which exhibited a median fragment size of less than 1 cm. In 20 cases, the incorporation of metric features enabled the validation of the morphological sex estimation through the implementation of the Bayesian model. In six cases, the morphology was ambiguous and metrics provided a more accurate sex estimation. The results of the metric and Bayesian sex estimation only deviated from each other in two cases. The morphological sex estimation was suspected to be less reliable due to features with a low degree of sexual dimorphism. Although the model was developed based on several Italian samples, which varied in terms of location and chronology, it demonstrated a high degree of accuracy. The findings from the Austrian sample indicate minimal interpopulation variations when compared from the Italian training sample. Further validation in different populations is necessary to ensure the findings can be generalized more widely.

We encourage our colleagues to use and critically validate our software further as well as to provide feedback, as we are planning to share updated versions in the future. It has been demonstrated that larger datasets, especially those containing data from various populations, may also lead to improved universal sex predictions over different time periods and geographical areas. We also encourage our colleagues to contact us if they have published metric data available that is ready to share. We are especially interested in receiving data that provides population-specific sex estimates.

## Conclusion

In this study, we presented a Bayesian model for metric sex estimation in human cremated remains based on postcranial features derived from a previous work [25]. Tested on the same sample, the Bayesian model was found to be more flexible than binary logistic regression modeling and simple cut-off points in handling missing data. Furthermore, the accuracies of these methods were comparable to those of postcranial sex estimation in dry bone material. A validation of the model using an independent sample from Austria sexed by morphological observations suggested that, in cases of discrepancy between morphological and metric estimations, the reliability of the morphological features may be uncertain. The results of the Bayesian model were found to be more reliable. The Bayesian model was integrated into an open access software application to facilitate its use in osteological research.

## Supporting information

S1 Data Raw data used for the Bayesian model. (CSV)

S1 File Supporting Information. Accuracies of the original cut-off point method and the binary logistic regression models. (PDF)

S1_Table Comprehensive overview of individuals from prehistoric Austrian samples used for the comparative analysis of sex prediction methodologies by three distinct metric approaches. Cut-off points (based on Cavazzuti, Bresadola [28]), single logistic regression models and Bayesian model) diagnosis are compared with the morphological sex determination, e.g., morphological features at the skull and pelvis. Single probabilities are provided for the regression model (in the event that multiple measurements were obtained, the probability for the most suitable model was utilized), as well as the Bayesian model. Furthermore, the table incorporates warnings derived from the Bayesian model to facilitate comprehension. The estimated of sex followed the following classification system; male/female (M/F, p ≥ 90), probable male/female (M?/F?, 80 ≤ p < 90), possible male/female (M??/F??, 65 ≤ p < 80), ambiguous (A, 50 ≤ p < 65).
(PDF)

S2 File Checklist. (PDF)
