# Supplementary material for: CRISP: Cremated remains inference of sex probabilities – A software for Bayesian sex estimation in human cremated remains
Source: PLoS One. 2026 May 5;21(5):e0346813. doi: 10.1371/journal.pone.0346813 (PMC13143051; doi:10.1371/journal.pone.0346813)
Supplement: S1 File — (PDF) [file pone.0346813.s002.pdf]

S2: Accuracies for the cut-off point method and the binary logistic regression models

The following table presents the accuracies per variable for the cut-off point method as published in Cavazzuti et al. (2019) and the binary logistic regression models. The latter method was further characterized by the calculation of its sensitivity and specificity (S2_Table 1). S2_Figure 1 displays the probability curves for the individual variables, along with the corresponding ROC plots.

S2_Tab1: Accuracies of the original cut-off point method and the binary logistic regression models.

	**cut-off point method**	**binary logistic regression models**			**receiver operating characteristic (ROC)**
**variable**	accuracy	accuracy	sensitivity	specificity	area under curve (AUC)
Mandible: condyle width	83.5	82.4	82.4	82.4	89.4
Axis: ant.-post. diameter	67.8	68.1	81.5	50.0	79.2
Humerus: vert. head diameter	80.0	80.0	70.0	86.7	94.0
Humerus: trochlea max. diameter	73.1	71.0	61.5	77.8	83.8
Humerus: trochlea min. diameter	72.3	73.2	64.0	80.6	77.6
Humerus: capitulum max. diameter	78.9	76.9	75.0	78.6	89.0
Radius: max. head diameter	88.3	88.3	84.6	91.2	93.8
Lunate: max. width	78.3	75.0	75.0	75.0	89.1
Lunate: max. length	80.2	76.5	66.7	81.8	87.9
Femur: vert. Head diameter	81.2	76.0	70.0	80.0	88.7
Patella: max. height	69.2	85.7	75.0	92.3	96.2
Patella: max. width	86.0	73.7	57.1	93.3	83.3
Patella: max. thickness	74.0	73.3	50.0	86.8	74.9
Talus: max. length	78.2	76.5	66.7	81.8	87.9
Talus: trochlea length	83.2	79.2	70.0	85.7	95.0
Talus: trochlea width	71.7	70.2	50.0	82.8	81.8
Navicular: max. length	74.6	76.3	60.0	87.0	75.9
MT1: dorsoplantar width of the head	75.6	77.8	69.6	83.9	84.9
MT1: med.-lat. width of the head (log)	80.6	81.6	82.4	81.0	86.3

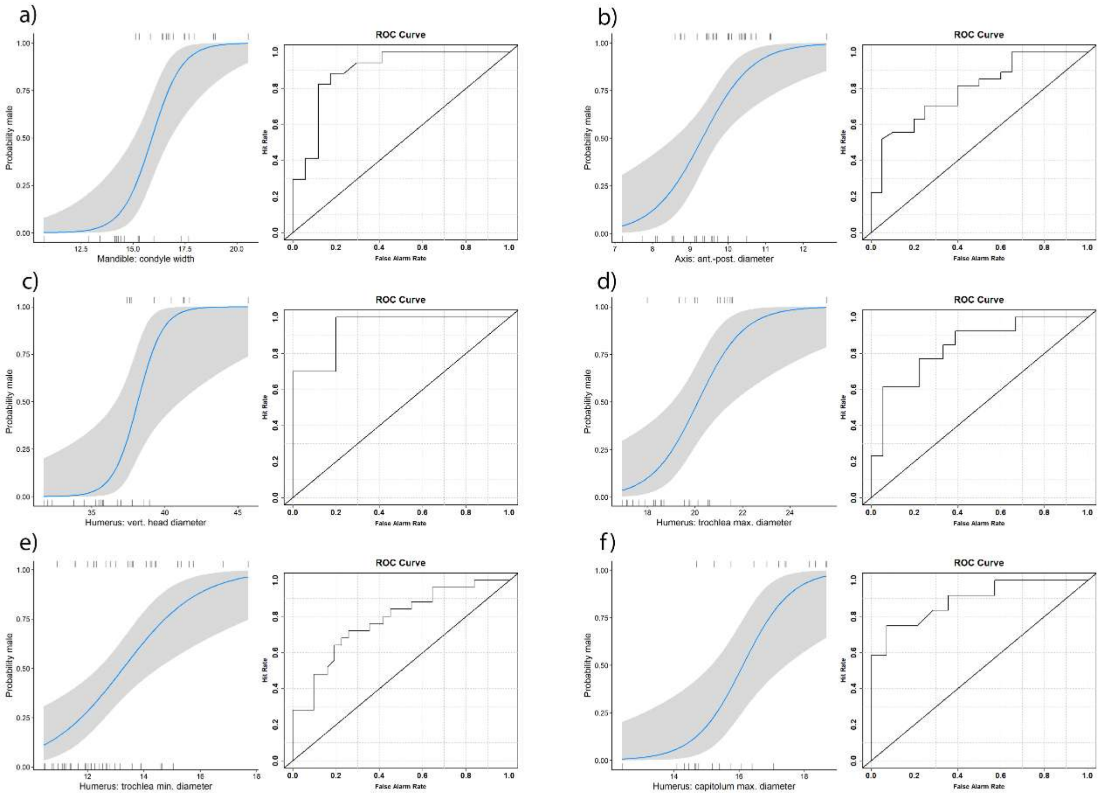

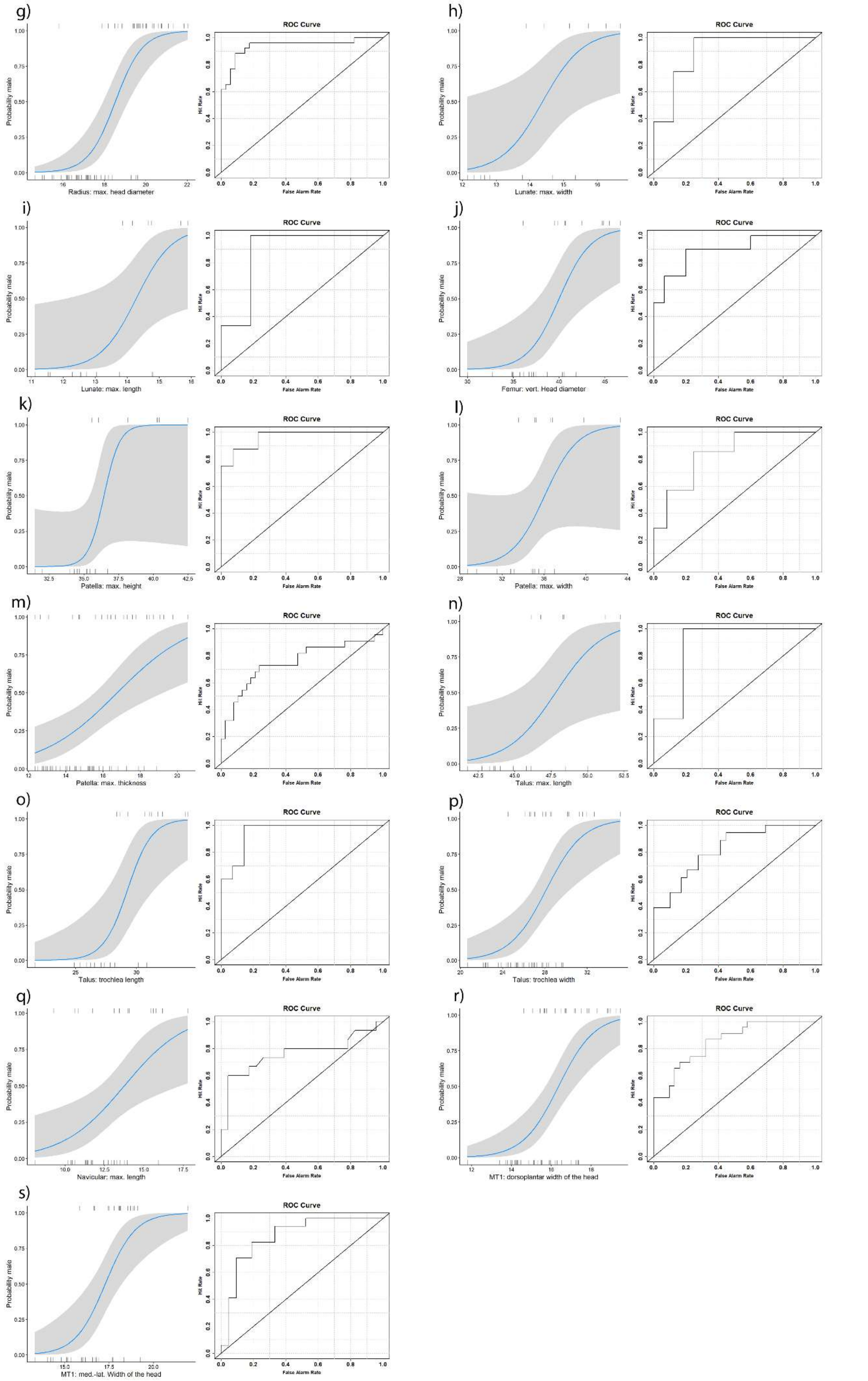

S2_Fig 1: Probability curves for sex prediction for all variables and corresponding ROC-plots: a) Mandible: condyle width, b) Axis: ant.-post. Diameter, c) Humerus: vert. head diameter, d) Humerus: trochlea max. diameter, e) Humerus: trochlea min. diameter, f) Humerus: capitulum max. diameter, g) Radius: max. head diameter, h) Lunate: max. width, i) Lunate: max. length, j) Femur: vert. Head diameter, k) Patella: max. height, l) Patella: max. width, m) Patella: max. thickness, n) Talus: max. length, o) Talus: trochlea length, p) Talus: trochlea width, q) Navicular: max. length, r) MT1: dorsoplantar width of the head, s) MT1: med.-lat. width of the head (log).
