# Supplementary material for: CRISP: Cremated remains inference of sex probabilities – A software for Bayesian sex estimation in human cremated remains
Source: PLoS One. 2026 May 5;21(5):e0346813. doi: 10.1371/journal.pone.0346813 (PMC13143051; doi:10.1371/journal.pone.0346813)
Supplement: S1_Table — Cut-off points (based on Cavazzuti, Bresadola [28]), single logistic regression models and Bayesian model) diagnosis are compared with the morphological sex determination, e.g., morphological features at the skull and pelvis. Single probabilities are provided for the regression model (in the event that multiple measurements were obtained, the probability for the most suitable model was utilized), as well as the Bayesian model. Furthermore, the table incorporates warnings derived from the Bayesian model to facilitate comprehension. The estimated of sex followed the following classification system; male/female (M/F, p ≥ 90), probable male/female (M?/F?, 80 ≤ p < 90), possible male/female (M??/F??, 65 ≤ p < 80), ambiguous (A, 50 ≤ p < 65). (PDF) [file pone.0346813.s003.pdf]

S3: Comparison of the sex prediction by metric and non-metric methods

S3_Tab1: Comprehensive overview of individuals from prehistoric Austrian samples used for the comparative analysis of sex prediction methodologies by three distinct metric approaches. Cut-off points (Cavazzuti et al., 2019), single logistic regression models and Bayesian model) diagnosis are compared with the non-metric sex determination, e.g., morphological features at the skull and pelvis. Single probabilities are provided for the regression model (in the event that multiple measurements were obtained, the probability for the most suitable model was utilized), as well as the Bayesian model. Furthermore, the table incorporates warnings derived from the Bayesian model to facilitate comprehension. The estimated of sex followed the following classification system; male/female (M/F, p ≥ 90), probable male/female (M?/F?, 80 ≤ p < 90), possible male/female (M??/F??, 65 ≤ p < 80), ambiguous (A, 50 ≤ p < 65).

site	grave	sex estimation								notes
		morphology	cut-off points	binary logistic regression			bayesian model			
					probability [%]			probability [%]		
					male	female		male	female	
Franzhausen-Kokoron	28	M	M	A	42.9	57.1	A	41.21	58.8	
Franzhausen-Kokoron	277	F?	F	A	35.1	64.9	A	36.21	63.8	
Franzhausen-Kokoron	291	F	A	A	63.3	36.7	A	44.92	55.1	
Franzhausen-Kokoron	326	M?	F	A	41.0	59.0	A	35.92	64.1	
Franzhausen-Kokoron	328	M?	M	A	51.0	49.0	A	52.57	47.4	
Franzhausen-Kokoron	366	M?	M	M	99.6	0.4	M	99.74	0.3	Inconsistency for few features | Δ = 0.497 | Posterior(m): rad_head_max: 0.497; hum_capit_max: 0.691; hum_vert_head: 0.994
Franzhausen-Kokoron	499	M?	F	F?	14.1	85.9	F??	21.42	78.6	
Franzhausen-Kokoron	505	M?	M	M??	85.2	14.8	M?	86.97	13.0	
Franzhausen-Kokoron	545	M?	M	M	92.3	7.7	M??	78.93	21.1	
Franzhausen-Kokoron	549	F?	A	A	63.2	36.8	F??	28.22	71.8	
Franzhausen-Kokoron	553	A	M	M??	66.6	33.4	M??	72.32	27.7	
Franzhausen-Kokoron	554	M?	M	M??	74.4	25.6	M	96.39	3.6	
Franzhausen-Kokoron	560	M??	F	A	36.7	63.3	F??	32.52	67.5	
Franzhausen-Kokoron	566	F??	M	A	58.0	42.0	A	61.02	39.0	
Franzhausen-Kokoron	576	A	M	A	50.0	50.0	A	55.19	44.8	
Franzhausen-Kokoron	584	F?	F	F?	10.6	89.4	F??	23.46	76.5	
Franzhausen-Kokoron	601	A	F	A	38.9	61.1	F??	34.18	65.8	
Franzhausen-Kokoron	605	M?	M	A	59.9	40.1	A	59.05	41.0	
Franzhausen-Kokoron	683	M	M	M	99.6	0.4	M	98.85	1.1	
Franzhausen-Kokoron	694	A	F	F	6.3	93.7	F	7.43	92.6	Inconsistency for few features | Δ = 0.336 | Posterior(m): MT1_DP_width: 0.096; rad_head_max: 0.432
Franzhausen-Kokoron	870	M?	M	M??	70.1	29.9	A	64.45	35.6	
Franzhausen-Kokoron	925	F	M	A	44.7	55.3	A	42.48	57.5	
Franzhausen-Kokoron	950	F??	M	M??	67.8	32.2	A	61.84	38.2	
Franzhausen-Kokoron	989	M	M	M	91.3	8.7	M	99.84	0.2	
Getzersdorf	4	M?	M	M	97.7	2.3	M	96.44	3.6	

Getzersdorf	7	F?	F	A	44.0	56.0	A	37.46	62.5	Inconsistency for few features | Δ = 0.335 | Posterior(m): patella_max_width: 0.275; patella_max_thick: 0.501; MT1_ML_width: 0.61
Getzersdorf	61	M?	F	A	40.8	59.2	A	50.15	49.9	
Getzersdorf	64	A	M	M?	85.4	14.6	M?	83.99	16.0	
St. Poelten	1	F	F	F	0.5	99.5	F	0.03	100.0	Inconsistency for several features!<br>→ Median Posterior(m): 0.395<br>→ deviation > 0.25 detected:<br>→ all individual posterior(m) values (ascending):<br>rad_head_max : 0.006 *<br>lunate_max_length : 0.067 *<br>femur_vert_head : 0.395<br>mand_cond_width : 0.496<br>axis_AP_diam : 0.543<br>* = deviation > 0.25
Inzersdorf	32-1	M?	M	M??	69.29	30.71	A	42.48	57.5	
Inzersdorf	97	A	F	F	8.59	91.41	F?	15.75	84.3	
Inzersdorf	163	M?	M	M?	89.5	10.50	M	94.63	5.4	
Inzersdorf	170-1	M	M	M	90.94	9.06	M	91.34	8.7	
Inzersdorf	181	M??	F?	F	0	100.00	M??	78.10	21.9	Inconsistency for few features | Δ = 0.426 | Posterior(m): hum_troch_min: 0.413; lunate_max_width: 0.492; rad_head_max: 0.839
Inzersdorf	189	F??	F	F	7.22	92.78	F?	11.80	88.2	
Inzersdorf	192	M??	A	A	47.18	52.82	M?	82.02	18.0	
Inzersdorf	193	F?	F	F	0.92	99.08	F	2.57	97.4	
Inzersdorf	197	M?	M	M??	73.61	26.39	M??	68.58	31.4	
Inzersdorf	217	M??	M	F	0	100.00	A	62.65	37.4	
Inzersdorf	219	F??	F	F?	14.55	85.45	F??	22.97	77.0	
Inzersdorf	222	M?	M	M	90.9	9.10	M??	68.71	31.3	
Inzersdorf	317	M?	M	M	100	0.00	M??	68.77	31.2	
